# Zika virus infection in pregnancy and infant growth, body composition in the first three months of life: a cohort study

**DOI:** 10.1038/s41598-019-55598-6

**Published:** 2019-12-16

**Authors:** Fernanda Soares, Andrea D. Abranches, Letícia Villela, Sarah Lara, Daniele Araújo, Sylvia Nehab, Leila Silva, Yasmin Amaral, Saint Clair G. Junior, Sheila Pone, Ludmila Lobkowicz, Nuria Sanchez Clemente, Patricia Brasil, Karin Nielsen-Saines, Marcos Pone, Elizabeth Brickley, Maria Elisabeth Moreira

**Affiliations:** 1 Instituto Fernandes Figueira/Instituto Nacional de Infectologia – Fundação Oswaldo Cruz, Rio de Janeiro, Rio de Janeiro, Brazil; 2 Department of Infectious Disease Epidemiology, London School of Hygiene and Tropical Medicine, London, UK; 3 Department of Epidemiology, University of São Paulo, São Paulo, Brazil; 4 Universidade da California –Pediatric Infectious Disease, UCLA, Los Angeles, California, USA

**Keywords:** Infectious diseases, Medical research

## Abstract

The implications of Zika Virus exposure in pregnancy for early infant growth remains poorly described. The main goal of this study is to compare the growth, body composition, and feeding modality of infants in the first three months of life by prenatal Zika Virus exposure status. We selected an analytical cohort of 115 infants born without microcephaly, comprising 56 infants with qRT-PCR confirmed exposure to ZIKV during gestation and 59 infants born to women with presumptively no evidence of ZIKV in pregnancy. Infants were evaluated at birth, 1 and 3 months of age in terms of anthropometrics, body composition All the results were adjusted by maternal age, maternal BMI and gestational age. We observe no differences between anthropometric measurements at birth. Mothers in exposed group showed higher BMI. At 1 month and 3 months of age there were differences in mid arm circumference, arm muscle circumference and fat free mass. Weight and length was less in the ZIKV exposed in pregnancy infants and statistically different at 3 month of age. The findings of this investigation provide new evidence that ZIKV exposure in pregnancy may be associated with differences in body composition.

## Introduction

In early 2015, Brazilian public health authorities first identified an increase in a “dengue-like illness”^1^, subsequently laboratory-confirmed to be caused by Zika virus (ZIKV)^1,2^. After its emergence in Brazil, ZIKV spread rapidly through the Americas, and, by March 2017, arthropod-borne transmission was reported in more than 80 countries worldwide^3^. While typically recognized to cause a mild and often asymptomatic presentation among adults^4^, vertical transmission of ZIKV in pregnancy has been associated with severe fetal consequences, leading to microcephaly^5,6^ and the wider Congenital Zika Syndrome (CZS) in affected neonates^7^. Although a series of recent prospective cohort studies has confirmed the links between ZIKV exposure in pregnancy and adverse birth outcomes and neurologic sequelae^8–11^, the impact of ZIKV infection in pregnancy on infant growth and body composition–and any potential mediating role of feeding modality–remains unknown.

We hypothesized that ZIKV infections in pregnancy may be associated with compromised infant development. Infections during pregnancy are not infrequent, and a growing body of literature has reported associations between prenatal exposure to infectious diseases, including human immunodeficiency virus (HIV)^12,13^ and malaria^14–16^, with childhood growth patterns. Similarly, it is also known that, among children with neurologic impairment, such as that associated with cerebral palsy, feeding difficulties are prevalent^17^ and may contribute to inhibited growth^18^. Indeed, several case series indicate that children with neurologic damage from congenital ZIKV infections may be at heightened risk of dysphagia^19,20^, arising from dysfunction in tongue movement and the pharyngeal phase of swallowing^21^.

To test this hypothesis, we followed up a subset of 115 infants without microcephaly who were participating in a prospective cohort study of children born during the ZIKV epidemic in Brazil. In comparing 56 infants with qtRT-PCR (Quantitative Reverse Transcription-Polymerase Chain Reaction) confirmed exposure to ZIKV during gestation with 59 infants born to women with no evidence of ZIKV in pregnancy, we aimed to improve understanding of how prenatal ZIKV exposure may influence the growth, body composition, and feeding modality of infants in the first three months of life.

## Methods

### Study design and participants

As part of an on-going prospective cohort study (clinicaltrials.gov NCT 03255369) based in Rio de Janeiro, Brazil, at the Instituto Fernandes Figueira of the Fundação Oswaldo Cruz (IFF-Fiocruz; http://www.iff.fiocruz.br/), the present work compares the growth, body composition, and feeding of infants exposed and unexposed to ZIKV during gestation. The analytical cohort includes new mothers and full-term neonates delivered at IFF-Fiocruz between June 2016 and September 2017 and excludes dyads whose infants were: (i) diagnosed with microcephaly (i.e., defined as a head circumference of <−2 standard deviations (SDs) (ii) diagnosed with chromosomal abnormalities (i.e., prenatally or at birth), (iii) diagnosed with a congenital infection from toxoplasmosis, rubella, cytomegalovirus, and herpes virus, or (iv) born to mothers with human immunodeficiency virus (HIV).

Maternal-child dyads were categorized by prenatal ZIKV exposure status. The exposed group comprised new mothers and neonates with symptomatic (i.e., presenting with exanthema, fever associated with arthralgia, myalgia, non-purulent conjunctivitis, and/or headache) and quantitative reverse transcription polymerase chain reaction (qRT-PCR)-confirmed ZIKV infections during pregnancy. The unexposed group comprised new mothers and neonates for whom there was no evidence of ZIKV infection during pregnancy (i.e., defined by the lack of maternal symptoms in pregnancy. As we did not have a good serological test to prevent the inclusion of exposed children from mothers who had asymptomatic infection, we included only mothers who had good adherence to mosquito bite prevention strategies- use of repellents and appropriate clothing to be worn during the epidemic). These infants were classified with presumptively unexposed.

### Clinical evaluation

Participating infants were clinically evaluated by trained researchers at birth, 1 and 3 months of age. it was accepted a variation of 15 days in these ages. During the study visits, researchers measured the following anthropometric variables in order to evaluate growth and body composition using WHO (World Health Organization) standard procedures^22^: weight (g), length (cm), cephalic perimeter (cm), abdominal circumference (cm), mid-arm circumference (cm), and triceps skinfold (mm).

Additional body composition indicators (i.e., percentage body water, percentage body fat, fat mass (g), and fat-free mass (g)) were assessed using electric bioimpedance and air displacement plethysmography. For total body water evaluation using biompedance (Quantum BIA 101Q device, RJL Systems, Inc., Clinton Townships, Michigan, USA), the electrodes were fixed on the right hand and foot with at least a 3 cm distance between them. The arms were kept away from the trunk, and the legs were kept apart. To record the measurement, the newborn infant was comfortable and quiet. The value of the resistance was recorded to calculate body water. The equation that was used to calculate total body water was proposed and validated by Tang, *et al*. using the technique of dilution with water marked with oxygen 18 isotope^23^. This equation uses two anthropometric measures (i.e., weight and foot length) and resistance (R) measured by electrical bioimpedance. The percentage of fat and fat-free mass was obtained through air displacement plethysmography (Pea Pod® Infant Body Composition System, Life Measurement, Inc., Concord, CA), which provides a safe and valid method to assess body composition of a newborn^24,25^. The muscular circumference of the arm was calculated with the circumference measurements of the mid-arm (cm) and triceps skinfold (mm, using the formula proposed by Frisancho^26^: Muscle Circumference of the Arm = Circumference of the Mid-Arm - (3.14 x Triceps Skinfold/10).

Nutritional status was evaluated using Z-score indices of weight, length, and head circunference for gestational age and sex based on WHO published data^27^. Additional information was collected on infant feeding, including: frequency, volume, and type (i.e., breast or formula milk). The presence of signs of dysphagia were also investigated (Dysphagia was reported by families with hypersalivation and difficulty in swallowing and confirmed by clinical examination or videofluoroscopy).

None of the exams required sedation or food breaks for the infant. In case of crying or irritation, the maternal breast was offered for 10 minutes if the mother was present and available for breastfeeding. Otherwise, infants were provided with 2 ml of a 10% glucose solution.

### Laboratory procedures

All the mothers had positive Real-time reverse transcriptase-polimerase chain reaction (RT-PCR) assays for ZIKV were perfomed with the QuantiTect probe RT-PCR kit (Qiagen) at Fiocruz in the group considered exposed group. Mothers of infants in the not exposed group had no symptoms of Zika Virus during pregnancy and we try to use infants that was born before the outbreak. We collected blood from

### Statistical analyses

The results were adjusted by maternal BMI (body mass index), maternal age and gestational age at birth. All P values are from 2-sided statistical tests, with a significance level of 5%. All data were entered into the Epi Info (Centers for Disease Control and Prevention, Atlanta, GA, USA), and all analyses were performed using the IBM SPSS Statistics, version 21.0 (Portsmouth, Hampshire, UK).

### Ethics

The project “Vertical Exposure to Zika Virus and its consequences on the development of the newborn”, of which this study is part of, was approved by the Ethics Committee in Human Research of IFF/Fiocruz under CAAE 52675616.0.0000.5269 and registered as NCT 03255369. The study was conducted in accordance with the Declaration of Helsinki, the International Conference on Harmonization guideline for Good Clinical Practice, and the codes and regulations of Brazil regarding research on human subjects. Infants were included in the study after their legal guardian(s) signed the Free and Informed Consent Form. The funders of the study had no role in data collection, analysis, or interpretation.

## Results

The study population consisted of a total of 115 infants, of whom 56 (48.7%) were intra-uterus exposed to ZIKV and 59 (51.3%) were unexposed. Among the ZIKV-exposed group, women tested RT-PCR positive in 24.1% of cases in the first trimester of gestation, 64.8% in the second trimester and 11.1% in the third trimester. Maternal anthropometric and comorbidity characteristics during pregnancy are described in Table 1. ZIKV-exposed women were on average older than ZIKV-unexposed women (30.1 years versus 29.5 years) although this was not statistically significant. Women exposed to ZIKV had a significantly higher BMI (26.6 versus 24, p = 0.013) and were more likely to be overweight/obese (58% versus 33.3%, P < 0.044) than unexposed women. A higher proportion of ZIKV-exposed women had hypertension (20% versus 12%) and diabetes (9.1% versus 5.2%) during pregnancy compared to unexposed women, although this was not statistically significant.

**Table 1:** Baseline characteristics of participating maternal-child dyads (N = 106) recruited between June 2016 and September 2017 at the IFF-Fiocruz in Rio de Janeiro, Brazil.

		ZIKV-exposed Dyads (N = 56)	ZIKV-unexposed Dyads* (N = 59)	*P* value^a^
		Mean (SD) or N (%)		
Mother	Age, years	30.1 (6.6)	29.5 (7.2)	*0*.*686*
	Pre-pregnancy Body Mass Index, kg/m^2^	26.6 (5.9)	24.0 (4.8)	*0*.*013*
	Pre-pregnancy Weight			*0*.*044*
	Normal Weight, BMI (18.5–24.9)	21 (42)	34 (66.7)	
	Overweight, BMI (25–29.9)	16 (32)	10 (19.6)	
	Obese, BMI ≥30	13 (26)	7 (13.7)	
	Missing	6 (10.7)	8 (13.5)	
	Weight Gain in Pregnancy, kg	11.8 (6.3)	13.0 (4.8)	*0*.*280*
	Gestational Hypertension			*0*.*250*
	No	45 (80.3)	52 (88.1)	
	Yes	11 (19.7)	7 (11.9)	
	Gestational Diabetes			*0*.*329*
	No	51 (91.1)	56 (94.9)	
	Yes	5 (8.9)	3 (5.1)	
	Trimester of ZIKV Infection		—	—
	First	13 (23.2)	—	
	Second	35 (62.5)	—	
	Third	8 (14.3)		
Neonate, at Birth	Sex			*0*.*778*
	Male	29 (51.8)	29 (49.2)	
	Female	27 (48.2)	30 (50.8)	
	Gestational Age, weeks	38.5 (1.2)	39.1 (1.3)	*0*.*008*
	Premature			*0*.*235*
	No	54 (96.4)	59 (100)	
	Yes	2 (3.6)	0 (0)	
	Small for Gestational Age			*0*.*417*
	No	52 (92.8)	53 (89.8)	
	Yes	4 (7.2)	6 (10.2)	
	Low Birthweight			*0*.*473*
	No	54 (96.4)	58 (98.3)	
	Yes	2 (3.6)	1 (1.7)	

^a^*P* values are from independent sample t-tests and Chi-squared tests as appropriate.

*Presumptively unexposed infants.

ZIKV-exposed infants were born, on average, one week before ZIKV-unexposed infants (38.5 weeks versus 39.1 weeks, p < 0.008) but still within the definition of full-term (>37 weeks) (Table 2). The mean weight (3275 g versus 3230 g), lenght (48.2 versus 50.4) and head circumference (34.9 cm versus 34.5 cm) at birth of infants exposed and unexposed to ZIKV did not differ significantly.

**Table 2:** Adjusted mean difference in basic anthropometric indicators at 0, 1, and 3 months of age comparing infants exposed (N = 56) and unexposed (N = 59) to ZIKV in pregnancy.

	Birth				1 Month of Age			3 Months of Age		
	ZIKV-exposed Infants (N = 56)		ZIKV-unexposed Infants* (N = 59)	p-value	ZIKV-exposed Infants (N = 56)	ZIKV-unexposed Infants* (N = 59)	Mean difference (95% CI)^a^	ZIKV-exposed Infants (N = 56)	ZIKV-unexposed Infants* (N = 59)	p-value
	Mean (SD)				Mean (SD)			Mean (SD)		
Weight, g	3284 (442)	3243 (361)		0,59	5002 (757)	5227 (756)	0,26	**6174** (**772**)	**6714** (**787**)	**0**,**04**
Length, cm	48.2 (6.6)	50.4 (8.4)		0,08	56.4 (2.7)	57.4 (2.7)	0,15	**60**.**4** (**2**.**3**)	**62**.**9** (**2**.**4**)	**<0**,**01**
Head Circumference, cm	34.9 (1.7)	34.5 (2.0)		0,32	38.8 (1.6)	39.0 (1.6)	0,65	40.8 (1.5)	41.8 (1.5)	0,06

^a^Mean difference (95% confidence interval) comparing ZIKV-exposed infants relative to unexposed infants as estimated from a linear regression with adjustment for maternal age, maternal pre-pregnancy BMI, and gestational age at birth.

*Presumptively unexposed infants.

At the first infant follow-up, carried out during the first month of life, ZIKV-exposed infants exhibited no differences in length, triceps skinfold, body fat percentage, fat mass and fat free mas compared to unexposed infants (Tables 2 and 3). In addition, mid arm circumference and arm muscle circumference at 1 and 3 months of age were significantly lower among ZIKV-exposed compared to unexposed infants. However, when followed-up in the third month of life, the only variables that remained significantly different between exposed and unexposed infants were weight, length, mid arm circumference, arm muscle circumferences, fat free mass (Tables 2 and 3).

**Table 3:** Adjusted mean difference in body composition at 1 and 3 months of age comparing infants exposed (N = 56) and unexposed (N = 59) to ZIKV in pregnancy.

		1 Month of Age			3 Months of Age		
		ZIKV-exposed Infants (N = 56)	ZIKV-unexposed Infants* (N = 59)	p-value	ZIKV-exposed Infants (N = 56)	ZIKV-unexposed Infants* (N = 59)	p-value
Anthropometry	Abdominal circumference, cm	38.2 (3.0)	39.4 (3.0)	0,12	25.4 (5.7)	26.4 (5.8)	0,38
	Triceps Skinfold, mm	7.1 (2.3)	7.6 (2.3)	0,45	8.5 (1.8)	7.9 (1.8)	0,33
	Mid-arm Circumference, cm	**12**.**4** (**1**.**3**)	**13**.**7** (**1**.**3**)	**<0**,**01**	**13**.**8** (**1**.**0**)	**14**.**7** (**1**.**0**)	**0**,**01**
Body Composition	Body fat, %	20.5 (6.2)	22.6 (6.2)	0,19	25.1 (6.0)	26.9 (5.8)	0,61
	Body water, %	58.3 (3.9)	57.0 (3.9)	0,16	52.6 (3.5)	51.1 (3.6)	0,22
	Fat Mass, g	1045 (428)	1212 (427)	0,14	1559 (475)	1792 (483)	0,16
	Fat-free Mass, g	3792 (614)	4020 (613)	0,16	**4480** (**413**)	**4953** (**419**)	**<0**,**01**
	Arm Muscle Circumference, cm	**10**.**1** (**1**.**3**)	**11**.**3** (**1**.**3**)	**<0**,**01**	**11**.**1** (**0**.**8**)	**12**.**2** (**0**.**8**)	**<0**,**01**

^a^Mean difference (95% confidence interval) comparing ZIKV-exposed infants relative to unexposed infants as estimated from a linear regression with adjustment for maternal age, maternal pre-pregnancy BMI, and gestational age at birth.

*Presumptively unexposed infant.

Among ZIKV-exposed infants, 37.8% were receiving some formula milk within the first month of life (compared to only 8.1% of unexposed infants, p = 0.002) and by the third month of life, 48.3% of exposed infants compared to 22.2% of unexposed babies were receiving some formula milk (p = 0.038). We tested the relation between the use of milk formula and the percentage of fat free mass, mid arm circumference and arm muscle circumference adjusting by group of exposition and we didn´t verify differences statistically significant. (p > 0.05)

Among ZIKV-exposed infants, 17.9% presented symptoms compatible with dysphagia (choking, hypersalivation, and reflux) and none in the not-exposed infants.

## Discussion

The effects of vertical ZIKV transmission on infant growth and body composition remain unknown. The results of this study indicate that at three month of life, ZIKV-exposed neonates are significantly lighter, smaller and have a lower fat free mass compared to unexposed neonates.

Growth involves much more than just a quantitative increase in body mass. Growth is the result of a complex interaction between several factors, such as genetics, nutrition and the environment. Weight gain in growing infants and children is non-specific, and the increase in adiposity and accumulation of lean mass and bone mass are also non-specific^28^. Body composition is an auxiliary measure of growth that allows evaluation of the quality of the weight gain. The body composition profiles of unexposed neonates in this study were comparative to healthy term infants as reported by Field, *et al*.^29^, who analysed 160 healthy term infants who were exclusively breastfed for 6 months. According to a body composition reference curve proposed by these authors, it is expected that at 1 month, infants have approximately 18 to 19.4% body fat. In our cohort, ZIKV-exposed infants presented with 20,5% body fat at one month of age. However, we observed some improvement in growth and an increase of 20% in body fat composition from the first to the third month of life. We hypothesize that as ZIKV-exposed babies have more difficulties with feeding and swallowing, that this may have led to an earlier introduction of formula milk via bottle. As an increased consumption of artificial formula milk during infancy results in an increase in fat mass^30^, this may have produced the catch-up growth observed in our study in ZIKV-exposed infants between one and three months of age.

In the case of neonates congenitally exposed to ZIKV, it is still not known to what extent ZIKV alters the intrauterine milieu and what effects this could have on the growth and body composition of the developing fetus during pregnancy. Studies have demonstrated that both nutritional deprivation and excessive intake by the fetus may affect gene expression in the neonate and have deleterious effects on its overall health^31^. A study performed by Brasil *et al*.^32^ in Rio de Janeiro with 125 ZIKV-positive pregnant women demonstrated that 46% of the neonates presented with global adverse neurological outcomes, such as seizures, hypertonia, hyperreflexia, and varying levels of neurodevelopmental delay; these outcomes are not completely understood to date. In this study, 17.9% of ZIKV-exposed infants had symptoms compatible with an impaired swallow and reflux. Thus, a timely diagnosis and early nutritional intervention would benefit these infants in the short and long term.

Nutritional intervention is still somewhat controversial and should be based on an individual patient-centered risk-benefit analysis. There is a consensus that optimal neurodevelopmental support is the highest priority, and therefore, like in preterm neonates, the current nutritional policy for ZIKV-exposed neonates seems to involve enhancing protein and energy consumption to promote growth and improve cognitive function. Therefore, the early introduction of formula milk has become commonplace to try meet nutritional demands and support infants with swallowing difficulties or neurodevelopmental delay.

According to the World Health Organization, adequate infant feeding comprises the practice of breastfeeding until 6 months of age and, at this point, the timely introduction of complementary feeding^33,34^. Of note, there is no recommendation to discontinue breastfeeding in mothers with concomitant ZIKV infections or in infants prenatally exposed to ZIKV^34^. However, it is not known whether ZIKV-exposed infants have more difficulties with breastfeeding due to dysphagia, or whether the early introduction of formula milk and discontinuation of breastfeeding could affect the mother/infant bond in ZIKV-exposed infants. In addition, it is unclear whether the total energy requirements described in the literature for healthy infants are sufficient for the maintenance of adequate growth, and body fat composition of ZIKV-exposed infants. However, it is likely that, by promoting growth and optimal neurodevelopment, early nutritional intervention could modify the life history of a ZIKV-exposed child (reviewed in^35,36^).

This study had limitations. One of the limitations of this study is the possibility that some of the ZIKV-unexposed women were in fact exposed to ZIKV during pregnancy. This is due to the challenges of acute ZIKV diagnosis given the fact that the majority of infections are asymptomatic. Because a sufficiently sensitive and specific serological test is not available, we need to include infants whose mothers had a good adherence to methods to prevent the infection. Then, we classified them in presumptively unexposed children.

Long-term studies with clinical and nutritional follow-up are needed to develop standardized protocols for the care of ZIKV-exposed pregnant women and their neonates. These would aim to promote and optimize both intrauterine and postnatal growth and minimize the risk of comorbidities, thereby stimulating maximum neurodevelopmental potential.

Clinical Trials Registry: NCT 03255369.
